# A Novel Splicing Mutation Alters DSPP Transcription and Leads to Dentinogenesis Imperfecta Type II

**DOI:** 10.1371/journal.pone.0027982

**Published:** 2011-11-18

**Authors:** Jun Zhang, Jiucun Wang, Yanyun Ma, Wenqi Du, Siyang Zhao, Zuowei Zhang, Xiaojiao Zhang, Yue Liu, Huasheng Xiao, Hongyan Wang, Li Jin, Jie Liu

**Affiliations:** 1 Department of Digestive Diseases, Fudan University, Huashan Hospital, Shanghai, China; 2 Ministry of Education (MOE) Key Laboratory of Contemporary Anthropology and State Key Laboratory of Genetic Engineering, School of Life Sciences, Institutes of Biomedical Sciences, Fudan University, Shanghai, China; 3 Department of Immunology, Shanghai Medical School, Institutes of Biomedical Sciences, Fudan University, Shanghai, China; 4 Department of Stomatology, Huashan Hospital, Fudan University, Shanghai, China; 5 National Engineering Center for Biochip Shanghai, Shanghai, China; University of Texas School of Public Health, United States of America

## Abstract

Dentinogenesis imperfecta (DGI) type II is an autosomal dominant disease characterized by a serious disorders in teeth. Mutations of dentin sialophosphoprotein (DSPP) gene were revealed to be the causation of DGI type II (DGI-II). In this study, we identified a novel mutation (NG_011595.1:g.8662T>C, c.135+2T>C) lying in the splice donor site of intron 3 of DSPP gene in a Chinese Han DGI-II pedigree. It was found in all affected subjects but not in unaffected ones or other unrelated healthy controls. The function of the mutant DSPP gene, which was predicted online and subsequently confirmed by *in vitro* splicing analysis, was the loss of splicing of intron 3, leading to the extended length of DSPP mRNA. For the first time, the functional non-splicing of intron was revealed in a novel DSPP mutation and was considered as the causation of DGI-II. It was also indicated that splicing was of key importance to the function of DSPP and this splice donor site might be a sensitive mutation hot spot. Our findings combined with other reports would facilitate the genetic diagnosis of DGI-II, shed light on its gene therapy and help to finally conquer human diseases.

## Introduction

Dentinogenesis imperfecta (DGI), a kind of hereditary dentin defect, causes the teeth of the patients discolored, translucent and even weaker than normal. It is classified into three types. DGI type I (DGI-I) is syndromic and mainly found simultaneously with osteogenesis imperfecta (OI). DGI type II (DGI-II) is more common and inherited in an autosomal dominant manner. Patients with DGI-II exhibit an opalescent hue of their discolored and translucent teeth. The teeth could be shortened approaching to the DEJ, even to the alveolar ridge. Narrow or even obliterated pulp chamber and root canals were shown under X-ray. DGI type III (DGI-III), which was first found in three isolated pedigrees in the United States in 1957, is now considered as a more severe form of DGI-II [1].

By linkage analysis and further sequence scan, the dentin sialophosphoprotein (DSPP) gene was identified to be the key gene associated with DGI, especially type II and type III [1]. DSPP gene is located on chromosome 4q21.3 and encodes the major noncollagenous protein in tooth dentin. Abundant insoluble mutant DSPP and its degradation products (dentin phosphoprotein, DPP and dentin sialoprotein, DSP) expressed by odontoblast cells could possibly be hypothesized to deactivate or indirectly interfere with the metabolism of other proteins in dentin and result in the phenotypes of DGI.

Mutations in the DSPP gene have been revealed and reported to be the principal cause of DGI type II and type III [1]–[4]. They could be in exons or introns, could affect one single peptide or cause frameshift of the entire following sequence [5]. Most of these mutations were just reported in DGI affected individuals by sequence analysis, but not further functionally confirmed. Functional confirmation of disease-causing or susceptible variations will definitely help in the understanding of the mechanism of the causation or susceptibility and solidify the association.

## Results and Discussion

**Figure 1:**
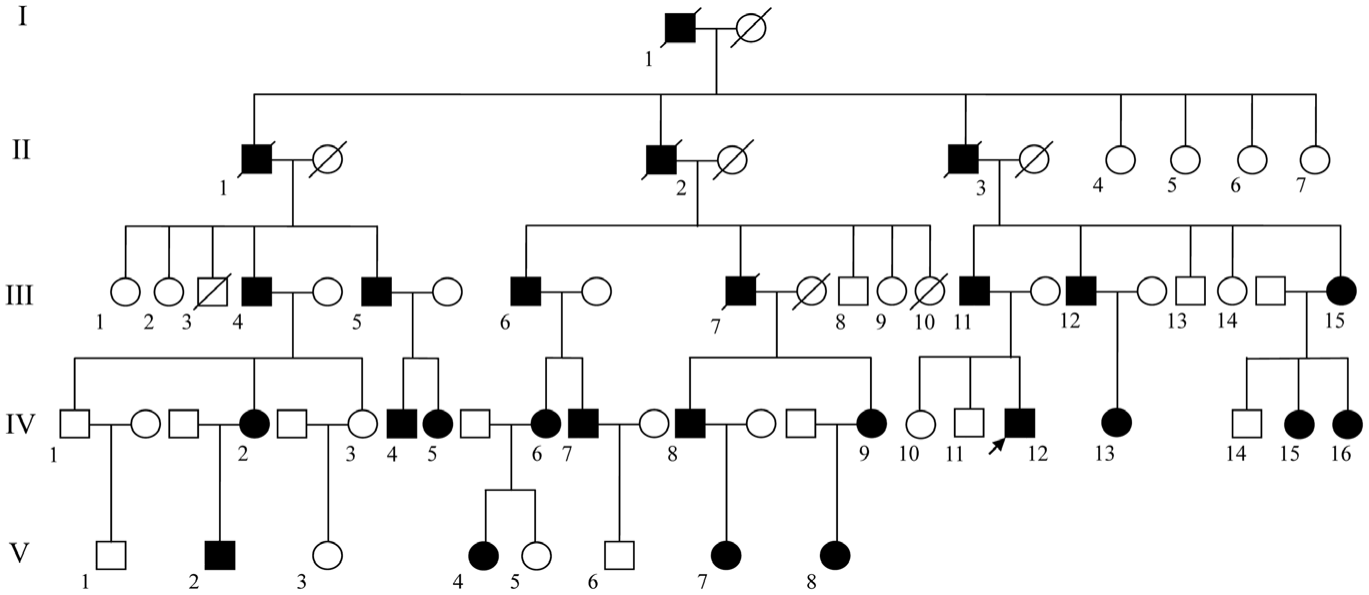
The family tree of the pedigree. The arrow shows the proband IV_12_.

**Figure 2:**
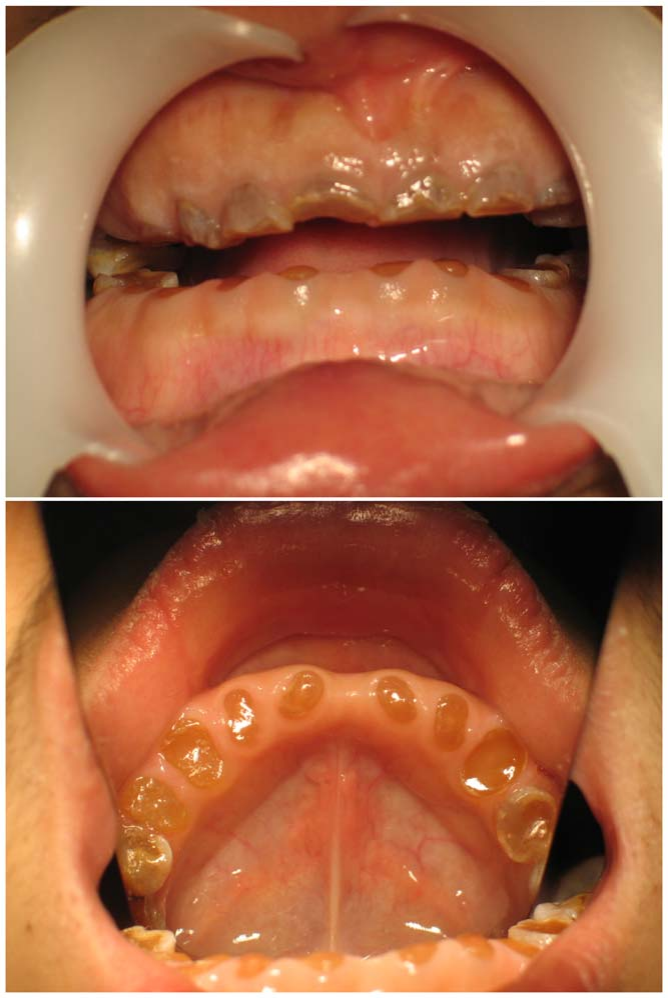
Clinical manifestations of the proband IV_12_.

We identified a pedigree with severe DGI-II in Shaanxi, China. From the family tree spanning five generations (Figure 1), the disease was shown to be inherited in an autosomal dominant manner. The proband is a young man, IV_12_ in the family tree. He was hospitalized because of dyspepsia. He presented with a severely affected permanent dentition. His all incisor teeth and four first molar teeth exhibited translucent and opalescent hue, color in dark-yellow to amber. The enamel on the occlusal and lingual surfaces was of serious wear. Dental crowns were shortened and globose, approaching to the DEJ, even to the alveolar ridge (Figure 2).

**Figure 3:**
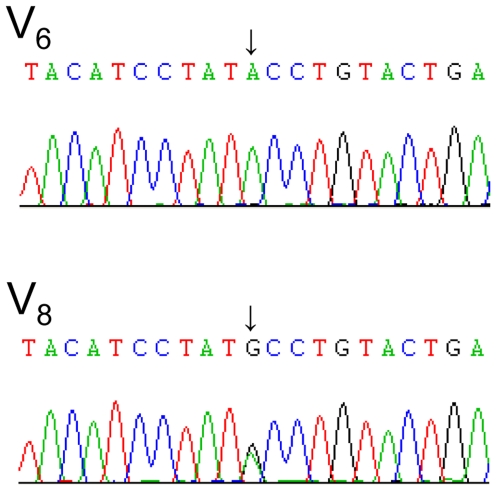
Sequencing raw data of subjects V_6_ (an unaffected member) and V_8_ (an affected member). The data are from the reverse complementary sequencing with the primer DSPP_New3R. The arrows show the complementary nucleotide of g.8662 site. V_6_ has homozygous A while V_8_ has heterozygous A/G.

The regions containing exon2, exon3 and intron3 of DSPP gene were sequenced to detect the causative mutation of this pedigree. Fortunately, a novel mutation in splice donor site of intron 3 (NG_011595.1:g.8662T>C, c.135+2T>C) was discovered in all affected subjects, while in four unaffected individuals and other 105 unrelated healthy controls, this site was in wild type (Figure 3).

Online splicing prediction by ***SplicePort*** (http://spliceport.cs.umd.edu) [6] showed that this mutation might change gene splicing by missing the normal splice donor (location: 3660, short sequence: tacaggtatagg, score: 0.938934), which might lead to the loss of splicing of intron 3. As no DSPP transcript was able to be detected in gingiva or peripheral blood sample by RT-PCR, we decided to confirm the function of this causal mutation by *in vitro* splicing analysis using the vector pLRT which contained the splicing reporter LTR-SD1-SAExon-SD-SA5opt [7], [8].

**Figure 4:**
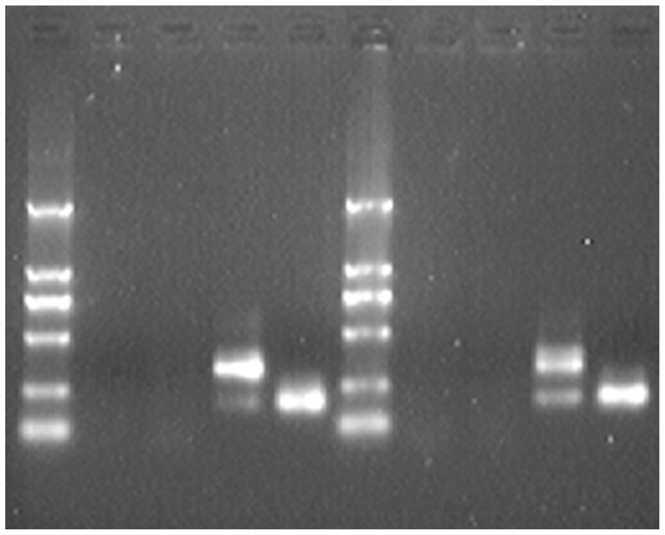
PCR products of DSPP alleles in splicing vector pLRT transfected into 293T and COS-1 cells. From left to right: D2000 marker, 293T cell control, naïve pLRT, mutant DSPP allele and wild type DSPP allele in 293T cells, D2000 marker, COS-1 cell control, naïve pLRT, mutant DSPP allele and wild type DSPP allele in COS-1 cells.

Both wild type and mutant DSPP alleles (1933bp containing whole exon3-intron3-exon4 and partial intron 2 and intron 4) were amplified and cloned into splicing vector pLRT to construct the recombinant plasmids. The plasmids together with naïve pLRT were transfected into 293T cells. Twenty-four hours after the transfection, total RNAs were extracted and reverse transcribed to cDNAs. By PCR and subsequent gel electrophoresis, difference in PCR product length was found between the cells transfected with wild type and mutant DSPP alleles. The product of wild type was 186bp in length, while the mutant had a main product of 319bp (Figure 4) indicating the non-splicing of intron 3. The products were further confirmed by sequencing from both strands. The *in vitro* splicing analysis was replicated by transfecting the plasmids into COS-1 cells. PCR result was the same as that in 293T cells (Figure 4). Thus, the splicing alteration of this mutation (non-splicing of intron 3) was proved. Taken together with the information from the family tree, this autosomal dominant splicing mutation g.8662T>C was indicated to be the causation of DGI-II in this pedigree.

Splicing alteration is an important disease-causing mechanism. It is reported that approximately 15% of human genetic diseases are caused by mutations at or near splice sites [9]. Previous studies have revealed several mutations (g.8658C>T [10], [11], g.8661G>A [12], g.8661G>T [5] and g.8663A>G [13]) in or adjacent to this splice donor site to be the causation of DGI-II. However, the function was not confirmed in any of these mutations. Instead, a skip of exon 3 (p.V18_Q45del) was hypothesized. In this study, we identified the novel causative mutation g.8662T>C in this site and for the first time confirmed its function, which was the non-splicing of intron 3. Before that, as to the function of DSPP mutations, only a skip of exon 3 was noticed by *in vitro* splicing analysis in another DSPP mutation [1]. Our results indicated that splicing was of key importance to the function of DSPP and that this splice donor site might be a sensitive mutation hot spot of DGI-II (another “hot spot” was reported as g.6191G>T (c.52G>T) [11], [12], [14]).

Genetic testing is a new method helping to predict, screen and diagnose diseases. Our results, combined with other reported data [5] offered DSPP single nucleotide mutations as well as the mechanism in DGI pedigrees. The findings would facilitate the genetic diagnosis of DGI through genetic testing, shed light on its gene therapy and help to finally conquer human diseases.

In conclusion, we identified a novel mutation in the splice donor site of DSPP (g.8662T>C), for the first time proved its function in altering gene transcription (non-splicing of intron 3), and concluded it to be the causation of DGI type II in a Chinese Han pedigree.

## Materials and Methods

### Ethics Statement

This study was approved by the Human Research Review Committee of Huashan Hospital, Fudan University. All participants signed the informed consent forms and all investigations were carried out adhered to the principles in the Declaration of Helsinki.

### Subjects

The pedigree was taken notice of in our clinical work. Five generations were included and three generations are living (III to V). 38 family members were visited (III_1_∼III_15_, IV_1_∼IV_16_, V_1_∼V_8_) and 26 DGI-II affected members were discovered (I_1_, II_1_, II_2_, II_3_, III_4_, III_5_, III_6_, III_7_, III_11_, III_12_, III_15_, IV_2_, IV_4_, IV_5_, IV_6_, IV_7_, IV_8_, IV_9_, IV_12_, IV_13_, IV_15_, IV_16_, V_2_, V_4_, V_7_, V_8_, consisting of 15 males and 11 females). 5-10mL of peripheral blood sample from each of 20 family members was collected (III_4_, III_6_, III_11_, III_15_, IV_1_, IV_2_, IV_3_, IV_6_, IV_7_, IV_8_, IV_9_, IV_12_, IV_15_, IV_16_, V_1_, V_2_, V_4_, V_6_, V_7_, V_8_, consisting of 16 affected and four unaffected members). DNAs were then extracted by QIAamp DNA Blood Mini Kit (QIAGEN, Hilden, Germany). At the same time, 105 unrelated healthy subjects were recruited as genomic controls to evaluate the revealed mutations.

### Sequence analysis

The genomic regions of interest were amplified by PCR and subsequently analyzed by double strand sequencing to detect the mutations. The PCR and sequencing were performed with the following primers:

DSPP-11F: AGTGCTGAGCCTGGTGATG

DSPP-11R: CACAGATATCACATAAAGCCC

DSPP-NEW3F: CAAGCCCTGTAAGAAGCCACT

DSPP-NEW3R: TGCTTCCAGCTACTTGAGGTC

The sequencing raw data revealing the novel g.8662T>C mutation was deposited in GenBank (accession number: JN625214). The following primers were used to confirm the mutation in affected and unaffected subjects from the pedigree as well as in the other 105 unrelated healthy controls:

PCR primers: DSPP1933_L: TCTGGTCACGCCTCCTGTTC, DSPP1933_R: GCATCCTGGTGCTTAGATTCCTT

Sequencing primers: F. Re-seq.:TTGGCAGGTTCCTCAAAGCA, DSPP319_R: TTCCCTCCTACTTCTGCCCAC

The sequencing raw data were read by the software Mutation Surveyor v3.24 (SoftGenetics, State College, United States) to reveal the variations.

The online splicing adaptor prediction was carried out by ***SplicePort*** (http://spliceport.cs.umd.edu). Both wild type and g.8662T>C substituted whole DSPP gene sequences were inputted and the prediction was performed using default settings.

### *In vitro* splicing analysis

The 1933bp DSPP allele containing whole exon3-intron3-exon4 and partial intron 2 and intron 4 was amplified by PCR with the following primers: DSPP1933_L_MunI: CCCCAATTGTCTGGTCACGCCTCCTGTTC and DSPP1933_R_XhoI: CCGCTCGAGCATCCTGGTGCTTAGATTCCTT. It was then cloned into the EcoR I/Xho I sites of pLRT to construct the recombinant plasmid. The plasmid was transformed into Escherichia coli strain DH5α to amplify and was subsequently extracted for further use.

Human Embryonic Kidney cell line 293T and African green monkey kidney fibroblast-like cell line COS-1 (Cell Resource Center of Shanghai Institutes for Biological Sciences, Chinese Academy of Sciences, Shanghai, China) were used in cell transfection. Twenty-four hours before transfection, 1.0×10^5^ cells were plated in each well of a 12-well plate. By adding 3 µL Lipofectamine 2000 (Invitrogen, Carlsbad, United States), 0.6 µg testing plasmid was transfected into the cells according to the manual of Lipofectamine 2000. The medium was changed to full Dulbecco's Modified Eagle Medium with high glucose (DMEM-h, Invitrogen) after four hours and the cells were harvested after 24 hours. Total RNAs of the cells were extracted by TRI reagent (Sigma-Aldrich, St. Louis, United States) according to the manufacturer's manual. Reverse transcription were carried out using High Capacity cDNA Reverse Transcription Kit (Applied Biosystems, Foster City, United States). cDNAs were then tested by PCR with the following primers: DSPP319_L: AAAATCCATGAATTTGCATCTCC and DSPP319_R: TTCCCTCCTACTTCTGCCCAC. The PCR product was evaluated by gel electrophoresis and confirmed by sequencing with the same primers.
